# Clinical Significance of Soluble Hemoglobin Scavenger Receptor CD163 (sCD163) in Sepsis, a Prospective Study

**DOI:** 10.1371/journal.pone.0038400

**Published:** 2012-07-20

**Authors:** Lin Feng, Xin Zhou, Long-Xiang Su, Dan Feng, Yan-Hong Jia, Li-Xin Xie

**Affiliations:** 1 Department of Respiratory Medicine, Hainan Branch of Chinese PLA General Hospital, Sanya, Hainan Province, China; 2 Department of Respiratory Medicine, Guangzhou Children’s Hospital, Guangzhou, China; 3 State Key Laboratory of Molecular Oncology and Department of Etiology and Carcinogenesis, Cancer Institute and Hospital, Chinese Academy of Medical Sciences and Peking Union Medical College, Beijing, China; 4 Department of Respiratory Medicine, Chinese PLA General Hospital, Beijing, China; 5 Department of Medical Statistics, Chinese PLA General Hospital, Beijing, China; University of Cape Town, South Africa

## Abstract

**Objective:**

We investigated serum soluble CD163 (sCD163) levels for use in the diagnosis, severity assessment, and prognosis of sepsis in the critical ill patients and compared sCD163 with other infection-related variables.

**Methods:**

During july 2010 and April 2011, serum was obtained from 102 sepsis patients (days 1, 3, 5, 7, and 10 after admission to an ICU) and 30 systemic inflammatory response syndrome (SIRS) patients with no sepsis diagnosed. Serum levels of sCD163, procalcitonon (PCT), and C reactive protein (CRP) were determined respectively. Sequential organ failure assessment (SOFA) scores for sepsis patients were also recorded. Then evaluated their roles in sepsis.

**Results:**

The sCD163 levels were 0.88(0.78–1.00)ug/mL for SIRS patients, 1.50(0.92–2.00)ug/mL for moderate sepsis patients, and 2.95(2.18–5.57)ug/mL for severe sepsis patients on day1. The areas under the ROC curves for sCD163, CRP, and PCT for the diagnosis of sepsis were, respectively, 0.856(95%CI: 0.791–0.921), 0.696(95%CI: 0.595–0.797), and 0.629(95%CI: 0.495–0.763), At the recommended cut-off 1.49 ug/mL for sCD163, the sensitivity is 74.0% with 93.3% specificity. Based on 28-day survivals, sCD163 levels in the surviving group stay constant, while they tended to gradually increase in the non-surviving group.The area under the ROC curve for sCD163 for sepsis prognosis was 0.706(95%CI 0.558–0.804). Levels of sCD163 with cut-off point >2.84 ug/mL have sensitivity of 55.8.0%, specificity 80.4%.Common risk factors for death and sCD163 were included in multivariate logistic regression analysis; the odds ratios (OR) for sCD163 and SOFA scores for sepsis prognosis were 1.173 and 1.396, respectively (P<0.05). Spearman rank correlation analysis showed that sCD163 was weakly, but positively correlated with CRP, PCT, and SOFA scores (0.2< r <0.4, P<0.0001), but not with leukocyte counts (r <0.2, P = 0.450).

**Conclusion:**

Serum sCD163 is superior to PCT and CRP for the diagnosis of sepsis and differentiate the severity of sepsis. sCD163 levels were more sensitive for dynamic evaluations of sepsis prognosis. Serum sCD163 and SOFA scores are prognostic factors for sepsis.

**Trial Registration:**

www.chictr.org ChiCTR-ONC-10000812

## Introduction

Sepsis is a systemic inflammatory disease that has a high incidence and mortality and requires high utilization rates of health care resources [1]. It is the second leading cause of death in the ICU after cardiac disease [2]. In the US, more than 500,000 patients are diagnosed with sepsis annually and their mortality is 35–45% [3]. Moreover, the mortality for severe sepsis patients is extremely high in which secondary multiple organ dysfunction (MODS) is the main cause of death [4]. An epidemiological study conducted in Europe showed that sepsis was promptly confirmed in only 37% of patients and that pathogens were identified in only 38.6% of these patients [5]. Although some variables, such as leukocyte counts, C reactive protein (CRP), and procalcitonin (PCT), have been applied to the diagnosis of sepsis and to determine its severity, recent evidence has highlighted the need for variables with high sensitivity and specificity that can be used to dynamically evaluate sepsis severity and prognosis.

**Figure 1:**
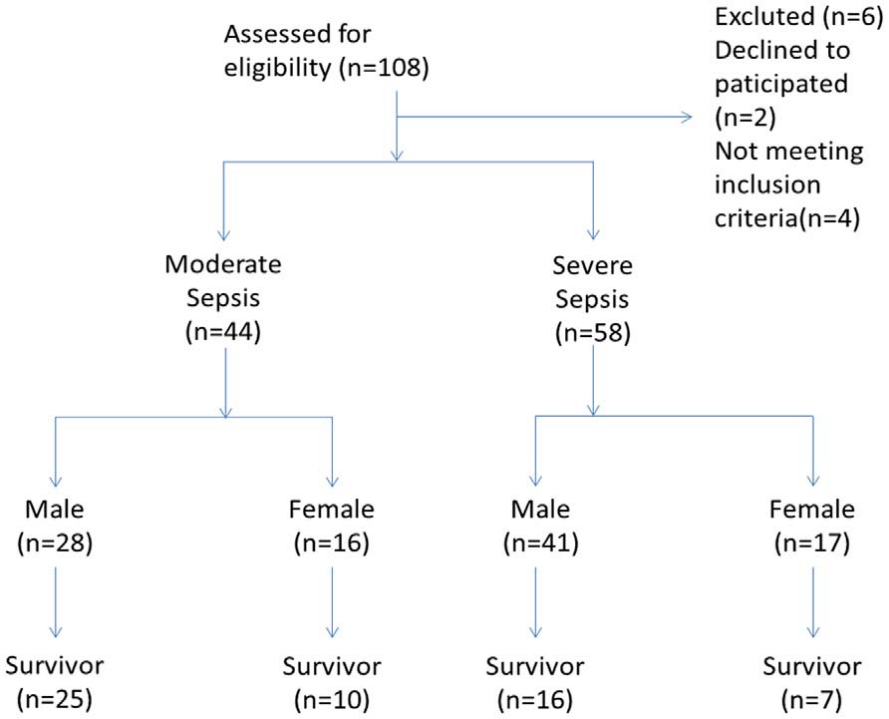
CONSORT flowchart,SURVIVOR means live more than 28 days.

**Table 1:** Patient baseline characteristics and outcomes.

	SIRS	Sepsis	*P* value^a^	Moderate Sepsis	Severe sepsis	*P* value^b^
Male	15	69		28	41	
Female	15	33		16	17	
Age Median+IQR	52±19	59±19	0.105	59±21	58±18	0.272
Mortality on day28,number(percentage)	1(3.33%)	44(43.14%)		9(20.45%)	35(60.34%)	
sCD163(ug/ml)	0.880.78–1.00	2.211.29–3.64	0.001	1.501.92–2.00	2.952.18–5.57	<0.001
CRP(mg/dl)	3.601.76–7.59	9.404.02–17.30	0.002	6.002.70–15.20	12.256.83–18.58	0.023
PCT(ng/ml)	0.380.20–1.96	1.50.22–17.30	0.114	0.530.14–4.63	2.590.86–12.00	0.024
WBC(×109/l)	11.559.50–12.64	11.278.11–15.90	0.824	10.598.15–13.33	12.048.01–17.06	0.252
SOFA		74–11		53–9	95–12	0.001
CRF		26		18	8	
CHF		22		10	12	
Hepatic insufficiency		7		1	6	
CKD		23		11	12	
immune suppression		16		5	11	

Results for age are given as means ± SDs. Results for all other variables are medians and IQRs. sCD163,soluble CD163;CRP, C-reactive protein; PCT, procalcitonin; WBC, white blood cell, SOFA, Sepsis-related Organ Failure Assessment; CRF, Chronic Respiratory Failure; CHF, Chronic Heart Failure; CKD,Cronic kidney dysfunction.

a Compared between patients with SIRS and sepsis.

b Compared between patients with moderate sepsis and severe sepsis.

CD163 is a unique hemoglobin receptor that is specifically expressed on the cell membrane of macrophages [6]. Soluble CD163 (sCD163) has been found to be related to several infectious and inflammatory diseases, although the underlying mechanisms are poorly understood [7]–[10]. Plasma levels of sCD163 are inversely associated with CD163 expression on macrophages [11]. In addition, CD163 plays an important role in innate immunity as it can recognize whole bacteria [7]. There is some evidence that oxidoreduction of hemoglobin and the subsequent release of inflammatory factors play critical roles in the pathogenesis of severe sepsis [12]. In this study, we investigated the use of sCD163 levels for the early diagnosis of sepsis and its prognosis for patients admitted to an ICU.

**Figure 2:**
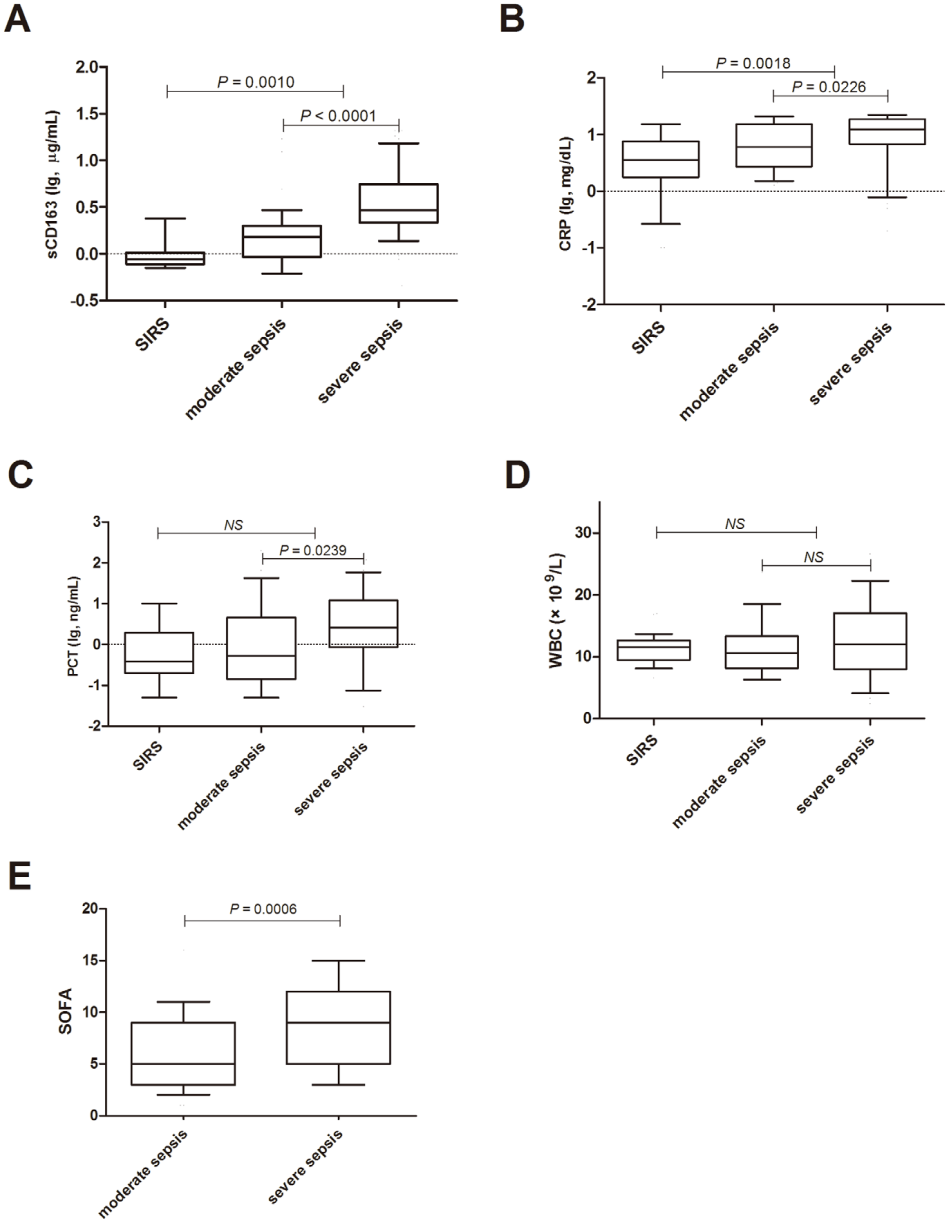
Boxplot of the levels of infective markers between SIRS and sepsis.

**Figure 3:**
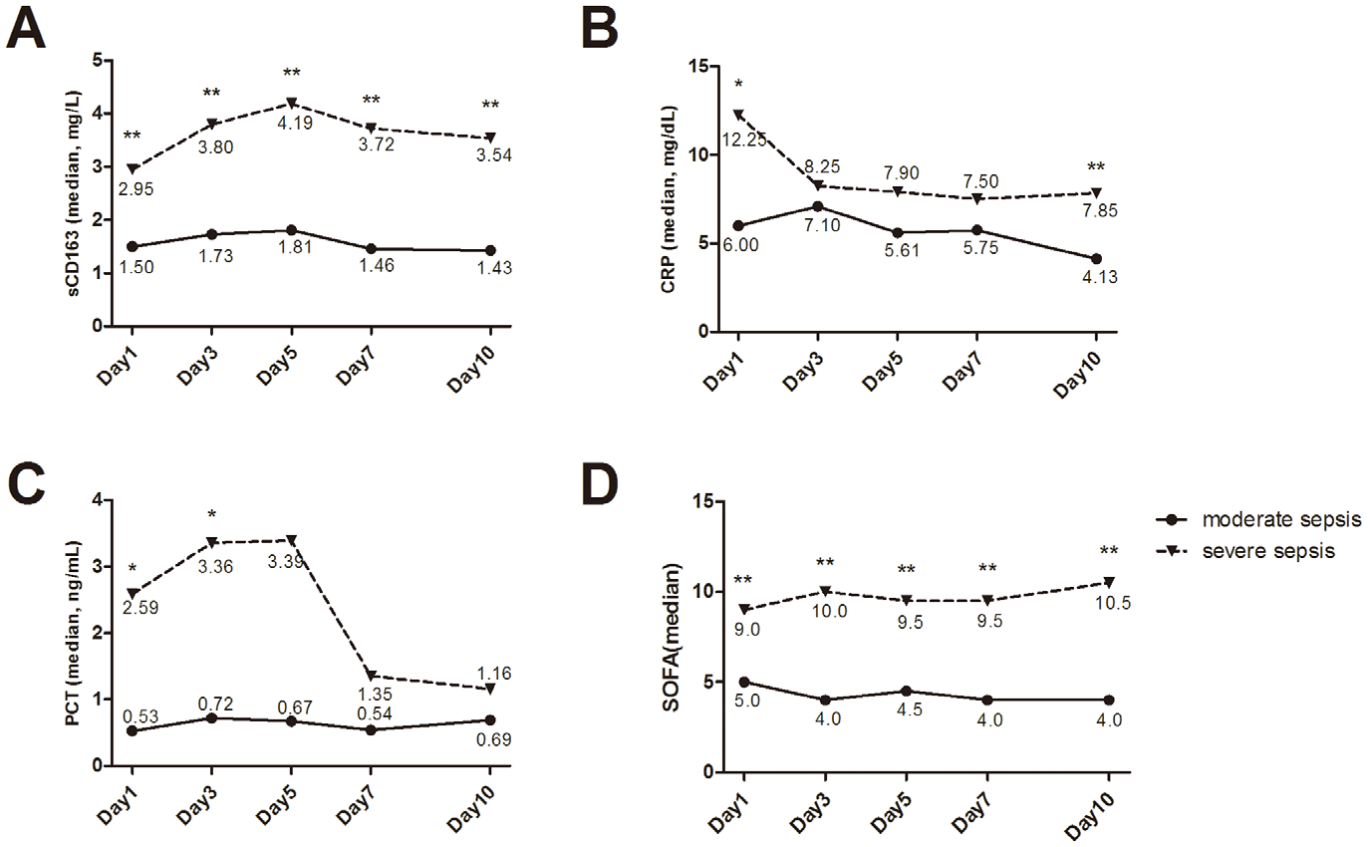
Dynamic changes of the mean value between moderate sepsis and severe sepsis, * means P<0.05, ** means P<0.01.

**Figure 4:**
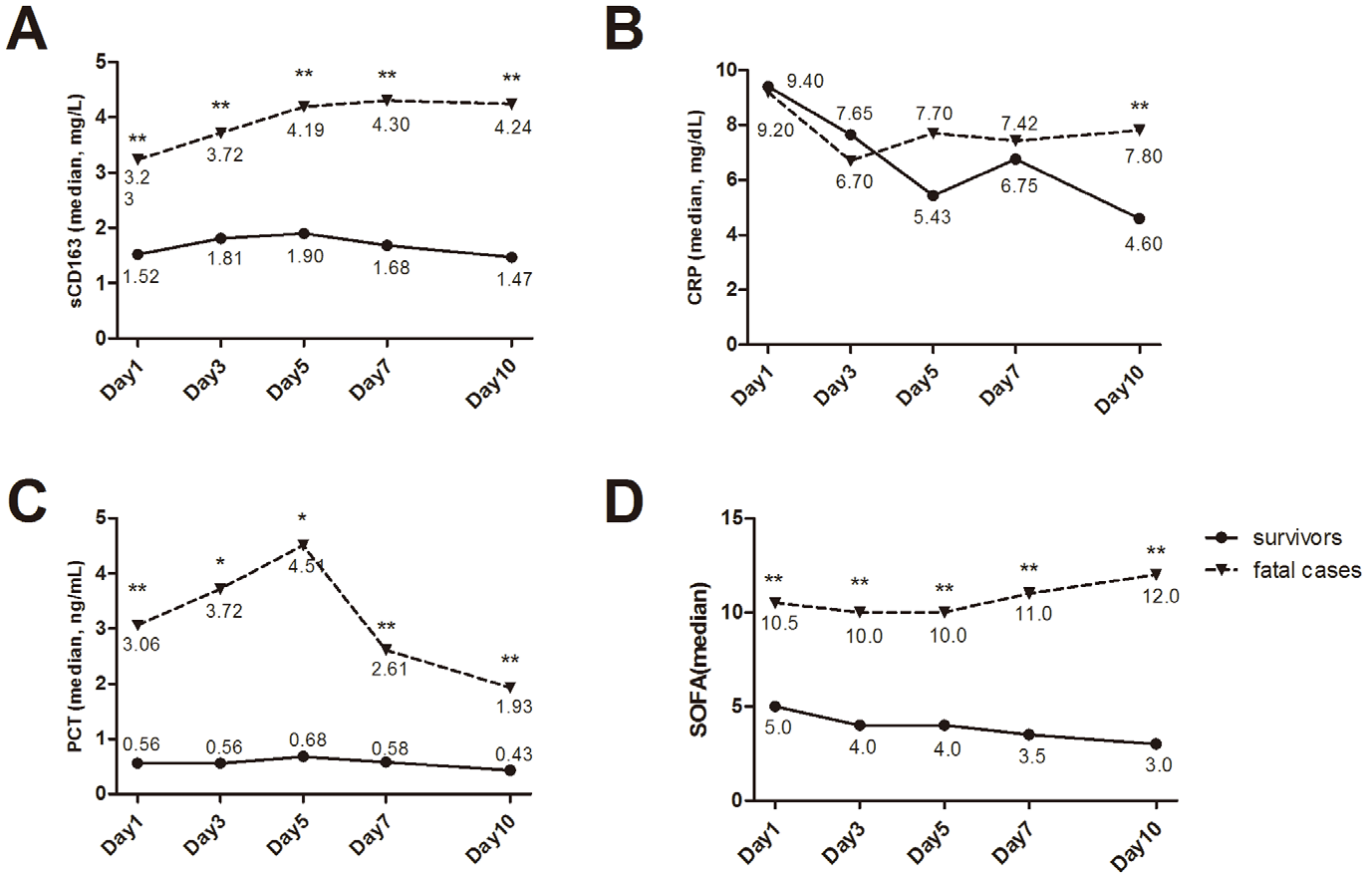
Dynamic changes of the mean between survivors and non-survivors, * means P<0.05, ** means P<0.01.

## Methods

### Patients

All subjects were selected from among inpatients who were hospitalized between July 2010 and April 2011 in the Respiratory Intensive Care Unit (RICU), Surgical Intensive Care Unit (SICU) and Emergency Intensive Care Unit (EICU), Chinese People’s Liberation Army General Hospital. The diagnosis of sepsis, the criteria used to distinguish between moderate and severe sepsis and the SOFA scores’ calculation were based on the ACCP/SCCM Joint Meeting criteria (1991).Sepsis is defined as the systemic inflammatory response syndrome (SIRS) with infection.More than one of the following clinical manifestations: (1) a body temperature greater than 38°C or less than 36°C; (2) a heart rate greater than 90 beats per minute; (3) tachypnea, manifested by a respiratory rate greater than 20 breaths per minute, or hyperventilation, as indicated by a PaCO_2_ of less than 32 mm Hg; and (4) an alteration in the white blood cell count, such as a count greater than 12×10∧9/L, a count less than 4×10∧/L, or the presence of more than 10 percent immature neutrophils. [13]. Exclusion criteria included: (1) patients younger than 18 years of age; (2) agranulocytosis (granulocyte counts <0.5×10^9^/L); (3) those discharged within 24 h; (4) those diagnosed with HIV infection; and (5) patients who refused to participate.

**Figure 5:**
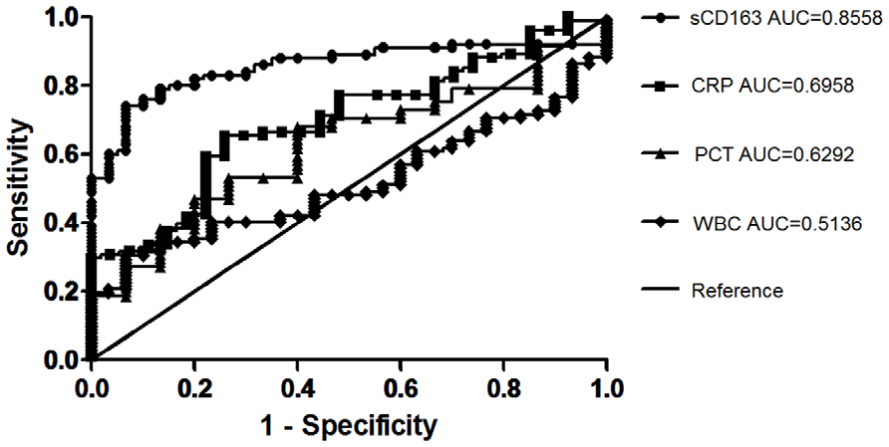
The receiver operating characteristic (ROC) curves comparing the discriminating capabilities of sCD163, CRP, PCT and WBC between SIRS and Sepsis.

**Table 2:** Areas under ROC curves (AUC) and 95%CI.

Markers	AUC^a^(95% CI)	Cutoff^a^ (sen%, sp%)	AUC^b^(95% CI)	Cutoff^b^ (sen%, sp%)
sCD163	0.856 (0.791,0.921)	1.49 ug/ml (74.00%,93.33%)	0.811 (0.723,0.899)	2.11 ug/ml (77.2%,79.1%)
CRP	0.696 (0.595, 0.797)	6.42 mg/dl (65.35%,74.07%)	0.633 (0.522,0.744)	9.5 mg/dl (48.3%,81.4%)
PCT	0.629 (0.495, 0.763)	0.55 ng/ml (67.90%,60.00%)	0.648 (0.522,0.774)	0.60 ng/ml (80.9%, 52.9%)
WBC	0.514 (0.415, 0.613)	13.8×109 (30.39%, 93.33%)	0.567 (0.455,0.678)	13.1×109 (43.1%, 75.0%)
SOFA			0.701 (0.599,0.803)	10 (48.5%, 81.4%)

a ROC curve analysis was used to distinguish between SIRS and sepsis;

b ROC curve analysis was used to distinguish between moderate and severe sepsis.

**Table 3:** Cut-off point and features of candidate diagnostic biomarkers.

	cutoff	Se (%)	Sp (%)	YI	PLR	NLR	PPV (%)	NPV (%)
Sepsis diagnosis								
sCD163^1^	1.49	74.0%	93.3%	0.673	11.1	0.279	97.4%	49.5%
sCD163^2^	1.01	80.0%	80.0%	0.600	4.00	0.250	93.2%	47.6%
CRP	6.42	65.4%	74.1%	0.394	2.52	0.468	89.5%	34.0%
PCT	0.55	67.9%	60.0%	0.279	1.70	0.535	85.2%	28.7%
Severe sepsis diagnosis								
sCD163	2.11	77.2%	79.1%	0.5626	3.69	0.289	82.9%	72.5%
SOFA	9.5	48.3%	81.4%	0.297	2.60	0.635	77.4%	54.4%
Prognosis of sepsis								
sCD163	2.84	55.8%	80.5%	0.362	2.84	0.550	68.2%	60.3%
SOFA	8.5	67.4%	73.2%	0.407	2.52	0.445	66.8%	58.1%
PCT	2.95	91.7%	44.4%	0.361	1.65	0.187	56.0%	41.8%

The time when patients were enrolled into the study was defined as the starting point. Venous blood (3 mL) was collected on days 1, 3, 5, 7, and 10. Blood was centrifuged at 3000 rpm for 15 min, serum was transferred to an aseptic tube, and stored at −80°C for later use.

**Figure 6:**
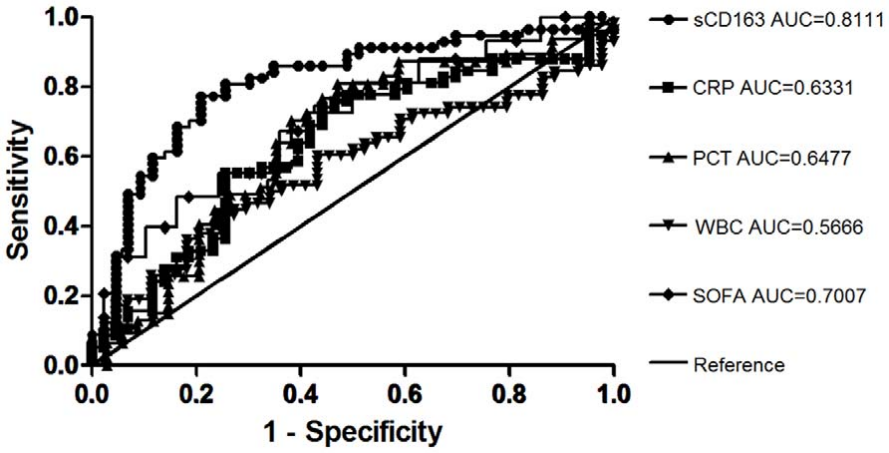
The receiver operating characteristic (ROC) curves comparing the discriminating capabilities of sCD163, CRP, PCT and WBC between moderate sepsis and severe sepsis.

This study was approved by the Ethics Committee of the General Hospital of PLA (20100701-002 ). Informed consent was obtained in written form before entry into the study from patients or their legal representatives.

**Figure 7:**
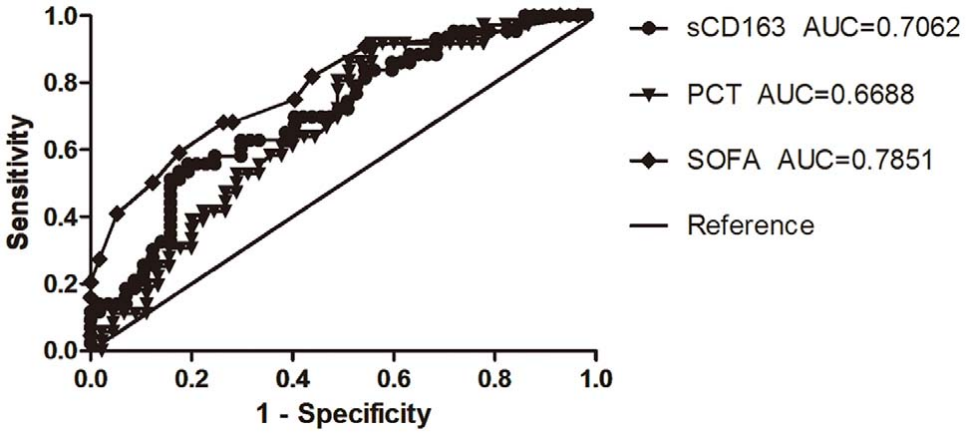
The receiver operating characteristic (ROC) curves determining sepsis prognosis of sCD163,PCT,SOFA.

**Table 4:** Areas under ROC curves (AUC) for predicting sepsis patients’ prognosis.

					95%CI	
	AUC	Cutoff	sen%	sp%	Lower bound	Upper bound
sCD163	0.706	2.84 ug/ml	55.8%	80.4%	0.604	0.808
PCT	0.669	8.5 ng/ml	67.4%	73.2%	0.551	0.786
SOFA	0.785	2.95	91.7%	44.4%	0.695	0.875

### Laboratory Tests

Serum sCD163 was determined using an ELISA kit (sCD163 kit; IQ, The Netherlands). CRP was determined by nephelometry using CardioPhase hsCRP (Siemens, Germany), and PCT was determined by ELFA using a VIDAS BRAHMS PCT kit (bioMerieux SA, France).

**Table 5:** Multivariate logistic regression analysis for sepsis prognosis.

						95%CI of OR	
	B	Standard Error	Wals c2	P value	Odd Ratio (OR)	Lower bound	Upper bound
CRF	−0.408	0.852	0.229	0.632	0.665	0.125	3.534
CHF	−0.597	0.789	0.572	0.449	0.551	0.117	2.585
CKD	0.835	0.789	1.121	0.2900	2.305	0.491	10.814
Hepaticinsufficiency	1.284	1.343	0.914	0.339	3.611	0.260	50.223
immune suppression	0.054	0.875	0.004	0.951	1.056	0.190	5.860
Gender	−1.221	0.766	2.538	0.111	0.295	0.066	1.325
Age	0.018	0.021	0.745	0.388	1.018	0.977	1.061
sCD163	0.159	0.078	4.155	0.042	1.173	1.006	1.367
T	−0.234	0.280	0.700	0.403	0.791	0.457	1.370
CRP	−0.029	0.046	0.388	0.533	0.972	0.8880	1.064
PCT	−0.009	0.011	0.705	0.401	0.991	0.970	1.012
WBC	−0.040	0.055	0.512	0.474	0.961	0.862	1.071
SOFA	0.334	0.097	11.740	0.001	1.396	1.154	1.690
Quantity	6.046	10.719	0.318	0.573			

CRF, Chronic Respiratory Failure; CHF, Chronic Heart Failure; CKD, Chronic kidney disfunction;T,Tempature;sCD163,soluble CD163;CRP, C-reactive protein; PCT, procalcitonin; WBC, white blood cell; SOFA, Sepsis-related Organ Failure Assessment.

### Statistical Analysis

Results for normally distributed variables are given as means ± standard deviations (SD), and results for non-normally distributed variables are given as medians and interquartile ranges (IQR). Group comparisons used Student’s t test for normally distributed variables and non-parametric Mann-Whitney tests for non-normally distributed variables. Categorical variables were compared with a Chi-square test. Receiver operating characteristic (ROC) curves were used to assess different variables with regard to the diagnosis and prognosis of sepsis and severe sepsis. Spearman rank correlation analysis was used to assess associations between variables. These analyses used GraphPad Prism v5.04. Risk factors, including 95% confidence intervals (CI), for 28-day survival were evaluated using multivariable logistic regression analysis incorporated in SPSS version 17.0. *P-*values <0.05 were considered statistically significant.

## Results

### General Clinical Characteristics

A total of 102 patients with sepsis and 30 patients with non-infection induced SIRS were enrolled in this study. Based on 28-day survivals, sepsis patients were divided into a surviving group (n = 58) and a non-surviving group (n = 44). Sepsis cases were categorized by severity into moderate sepsis (n = 44) and severe sepsis (n = 58); the mortality rate was 20.45% for moderate sepsis patients and was 60.34% for severe sepsis patients (Figure1).

Among the sepsis patients, there were 69 males (mean age = 60±20 years) whose mortality rate was 40.58%, and 33 females (mean age = 57±19 years) whose mortality rate was 48.48%. The mean age was 54±20 years for SIRS patients and 59±21 years for all of the sepsis patients.

Among the sepsis patients, concurrent chronic diseases included (1) chronic respiratory failure, (2) chronic heart failure, (3) chronic liver dysfunction, (4) chronic renal failure, and (5) immune suppression. The mortality rates among these patients with different co-morbidities were, respectively, (1) 38.5%, (2) 40.9%, (3) 71.4%, (4) 52.2%, and (5) 37.5%. There were no significant differences in terms of mortality among patients with these different co-morbidities (*P* = 0.491). In addition, some of these patients had more than one co-morbidity mentioned above (Table 1). Infections involved the lungs, blood, biliary system, peritoneum, urinary system, and others (skin, soft tissues, etc.). The mortality rate for patients with pulmonary infections was as high as 50.9%, which was the highest among patients with infections of different organs.

### sCD163 and Common Clinical Variables

On day 1, the serum sCD163 levels were 0.88 (0.78–1.00) µg/mL for SIRS patients, 1.50 (0.92–2.00) µg/mL for moderate sepsis patients, and 2.95 (2.18–5.57) µg/mL for severe sepsis patients. The serum sCD163 and CRP levels in sepsis patients were significantly higher than those in the SIRS patients (*P*<0.01), but no marked differences were observed for PCT levels and WBCs between these two groups.

The sCD163 levels in severe sepsis patients were markedly higher than those in moderate sepsis patients (*P*<0.0001), and similar trends were also observed for CRP and PCT levels and SOFA scores (Figure 2). In addition, sCD163 levels and SOFA scores in severe sepsis patients were remarkably higher than those in moderate sepsis patients at different time points (Figure 3). sCD163 levels in severe sepsis patients peaked on day 3 and then gradually declined, which may suggested that inflammation in the severe sepsis patients had reached maximum levels on day 3. However, the sCD163 levels in moderate sepsis patients did not markedly change over time. Moreover, the sCD163 levels and SOFA scores in the non-surviving group at different time points were significantly higher those in the surviving group.If the level was not declined, which meaned a worse prognosis for the patients (Figure 4). These dynamic changes indicated that serum sCD163 was a stable predictor of sepsis outcomes.

### Diagnosis of Sepsis and Severe Sepsis

ROC curve analysis was applied to the initial values of the several variables to assess their diagnostic performance. For the diagnosis of sepsis, the areas under the ROC curves for the initial levels of sCD163, CRP, and PCT were, respectively, 0.856 (95% CI: 0.791–0.921), 0.696 (0.595–0.797), which showed a more favorable performance for sCD163. Thus, sCD163 can be used for the early diagnosis of sepsis. At the recommended cut-off 1.49 ug/mL for sCD163, the sensitivity is 74.0% with 93.3% specificity (Figure 5, Table 2, Table3).

For the diagnosis of severe sepsis, the areas under the ROC curves for the initial sCD163, CRP, and PCT levels and SOFA scores were, respectively, 0.811 (95% CI: 0.723–0.899), 0.633 (0.522–0.744), 0.6477 (0.522–0.774), and 0.701 (0.599–0.802); The optimal cut off point was estimated to be about 2.11 ug/mL, at which the sensitivity and specificity of the test would be 77.2% and 79.1% this showed the that sCD163 was better than PCT, CRP, and SOFA scores for the diagnosis of severe sepsis. These results indicated that serum sCD163 was superior to other infection-related variables and SOFA scores for the diagnosis of either sepsis or severe sepsis (Figure 6, Table 2, Table3).

### Evaluation of Sepsis Prognosis

There were significant differences in the initial sCD163 and PCT levels and SOFA scores between the non-surviving and surviving groups. ROC curves were generated for these variables to evaluate their performance for disease prognosis. For sepsis prognosis, the areas under the ROC curves for initial sCD163 and PCT levels and SOFA scores were, respectively, 0.706 (95%CI: 0.604–0.808), 0.669 (0.551–0.786), and 0.758 (0.695–0.875), which showed that sCD163 levels and SOFA scores exhibited moderate performance for determining sepsis prognosis. Levels of sCD163 with cut-off point >2.84 ug/mL have sensitivity of 55.8.0%, specificity 80.4% (Figure 7, Table 3, Table 4).

### Logistic Regression Analysis

The initial values for sCD163, CRP, and PCT levels, WBCs, SOFA scores, age, gender, and co-morbidities were included in a multivariable logistic regression analysis. These results showed that sCD163 levels and SOFA scores were factors associated with disease prognosis with respective odd ratios of 1.173 and 1.396 (*P*<0.05; Table 5).

Correlations between sCD163 and other infection related variables.

Spearman rank correlation analysis showed that sCD163 levels were weakly, but positively associated with CRP levels (r = 0.317, *P*<0.0001), PCT levels (r = 0.360, *P*<0.0001), and SOFA scores (r = 0.365, *P*<0.0001). However, there was no correlation between sCD163 levels and WBCs (r = 0.037, *P* = 0.4501).

## Discussion

CD163 is a unique receptor for free hemoglobin in the blood. Blood levels of sCD163 have prognostic value for several inflammatory diseases and may have use in clinical applications as a biomarker of inflammatory diseases. In 2009, Fabriek *et al*. [7] found that sCD163 had innate immunity functions *in vitro* and that it could recognize whole bacteria. However, the *in vivo* roles for sCD163 and its related signaling pathways remain largely unknown. There may be a novel pathway involved with sCD163 activities, which may provide new strategies for treating certain diseases. In the present study, we evaluated the utility of serum sCD163 levels for the diagnosis and prognosis of sepsis. Our results showed that sCD163 had clinical value for sepsis diagnosis and prognosis.

In our study, on day 1 of their admission to an ICU, the serum sCD163 levels in sepsis patients were markedly higher than in SIRS patient, and were superior to common infection-related variables (CRP and PCT) for diagnosing sepsis. Therefore, sCD163 may be of value as a sensitive, specific diagnostic marker of infection. Our study also found that sCD163 was superior to CRP and PCT levels and SOFA scores for the evaluation of sepsis severity and for the dynamic monitoring of sepsis, which may be attributed to the pathogenesis of severe sepsis. Larsen *et al*. showed that an increase of free hemoglobin in the blood played an important role in the development of severe sepsis and can aggravate tissue injury [12]. In addition, ICU patients typically have co-morbid chronic diseases, which may decrease the sensitivity of SOFA scores.

Several studies have shown that serum sCD163 is closely related to the diagnosis and prognosis of several infectious diseases [7]–[10] and that its expression is regulated by some inflammatory factors [15]. In this study among sepsis patients, sCD163 levels in the non-surviving group were significantly higher than those in the surviving group and could be of certain value for determining sepsis prognosis. There is some evidence that an increased sCD163 level is a risk factor for a poor prognosis with bacterial infections for patients younger than 75 years of age [8]. In our study, serum sCD163 levels and SOFA scores were two factors associated with a poor prognosis for infection-induced sepsis. Thus, we postulate that sCD163 should be applied as a factor for the prognosis of infections. We also found that sCD163 was superior to CRP and PCT for the differentiation of sepsis. Although as an inflammatory biomarker, PCT has emerged as the most studied and promising sepsis biomarker. For diagnostic and prognostic purposes in critical care, PCT is an advance on C-reactive protein and other traditional markers of sepsis, but is not accurate enough for clinicians to dispense with clinical judgement [16]. Therefore, we need find more sensitive and specific biomarkers on sepsis diagnosis. Based on previous findings [8], [9] and those of this study, we postulate that sCD163 can be applied to monitor infection and act as a guide for the use of antibiotics in clinical practice. Our results also showed that sCD163 levels and SOFA scores were associated with the prognosis of sepsis, which was different from previously reported results [14]. This might be attributed to the small sample size of our study. Although the sample size was small, our findings still have certain clinical implications. Given the differences in sCD163 levels between our non-surviving and surviving groups, we found that an increase in sCD163 levels may predict a poor prognosis for sepsis patients.

Limitations of the present study are that it was a single-center trial and the sample size was small. More studies with a larger sample sizes are needed to confirm our results.

### Conclusion

The serum sCD163 level might have potential value on the diagnosis of sepsis and severe sepsis, and its performance is superior to PCT and CRP levels. sCD163 also would have advantages for the dynamic monitoring of sepsis development and prognosis and have favorable prospects for use in clinical applications for all that it need larger samples sizes of clinical study to confirm the value of sCD163 in sepsis.
